# Patients with symptoms and characteristics consistent with obstructive sleep apnea are at a higher risk for acute and subacute stent thrombosis after percutaneous coronary stent implantation: a single-center case–control study

**DOI:** 10.1186/s12872-017-0658-3

**Published:** 2017-08-18

**Authors:** Yanhua Li, Shanshan Yang, Si Chen, Xinhong Guo, Yundai Chen

**Affiliations:** 1 Department of Cardiology, Chinese PLA General Hospital, Beijing, 1000853 China; 2 Institute of Geriatrics, Chinese PLA General Hospital, Beijing, China; 3 Jinan Military Area CDC, Jinan, Shandong China

**Keywords:** Obstructive sleep apnea, Percutaneous coronary intervention, Conventional cardiovascular risk factors, Case–control study

## Abstract

**Background:**

To determine if obstructive sleep apnea (OSA) is a risk factor for early stent thrombosis (EST; within 30 days) after percutaneous coronary intervention (PCI).

**Methods:**

This case–control study involved 23 patients with angiographically confirmed EST after PCI (case group) and 92 PCI patients (control group) who did not develop stent thrombosis during a 2-year follow-up. Patients with symptoms and characteristics consistent with OSA (hereinafter referred to as OSA) were identified using the Berlin questionnaire, and the general characteristics of the patients and their treatments as well as outcomes were recorded. The odds ratios (ORs) for OSA were calculated. Additionally, the association between OSA and EST in patients with different conventional cardiovascular risk factors was analyzed.

**Results:**

The crude OR for OSA was 4.17 (95% confidence interval [CI]: 1.60–10.84, *P* = 0.003). After adjusting for other risk factors of EST, the OR for OSA remained significant. In participants with no or one conventional cardiovascular disease risk factor, we found a significant association between OSA and EST (OR: 17.00, 95% CI: 2.33–124.19, *P* = 0.005).

**Conclusion:**

OSA is an independent risk factor for EST. This conclusion was further supported by the finding that in patients with few conventional cardiovascular risk factors, the contribution of OSA to EST was more obvious.

## Background

Stent thrombosis is one of the most serious complications of percutaneous coronary intervention (PCI). Even with the use of newer antiplatelet agents, such as P2Y12 receptor inhibitors (e.g., prasugrel and ticagrelor) in combination with aspirin, the incidence of early stent thrombosis (EST) after PCI has been reported to be 1.4%–1.7% [1]. Although this incidence seems relatively low, EST is a life-threatening complication, as it often leads to acute myocardial infarction or sudden death, and is associated with a mortality rate of up to 45% [2]. Therefore, the risk factors for EST should be identified, and high-risk patients should be carefully monitored.

Obstructive sleep apnea (OSA) is very common in patients with cardiovascular diseases. The prevalence of OSA in patients with cardiovascular diseases has been reported to be 46% [3], while in patients undergoing PCI or coronary artery bypass graft, the prevalence of moderate-to-severe OSA is as high as 63.7% [4]. OSA is an independent risk factor for coronary heart disease, hypertension, atrial fibrillation and other arrhythmias, and stroke.[5–8] Moreover, it adversely affects patient prognosis. A recent prospective study [9] reported that OSA was associated with an increase in the rate of major adverse cardiac events (MACE) in patients who had undergone coronary stenting. The incidence of MACE after coronary stenting is much higher in patients with sleep apnea (25%) than in patients without sleep apnea (16%, *P* = 0.038, [10]. Additionally, most of the increase in the MACE rate was accounted for by an increase in the incidence of perioperative myocardial infarction. Considering the above reports, we hypothesized that OSA may be a risk factor for EST after PCI. We, therefore, conducted this case–control study to determine the association between OSA and EST in patients undergoing PCI.

## Methods

### Ethics approval

The study received ethical approval from the Committee for Medical Ethics of the Chinese PLA General Hospital, Beijing, China. Written consent was obtained.

### Study population

This case–control study involved 23 patients (18 men, 5 women) in the case group, who were diagnosed with acute or subacute stent thrombosis after undergoing PCI at our hospital between January 2010 and January 2015. The inclusion criteria for the case group were as follows: age greater than 18 years, successful PCI in at least one major coronary artery, and angiographically documented EST. Patients who could not provide information regarding OSA were excluded. Using age (±5 years) and gender as matching conditions and a ratio of 1:4, we enrolled 92 control patients who had been treated in the cardiology department during the same time period. All control patients had undergone stent surgery and had not experienced stent thrombosis during 2 years of follow-up.

### Measurements

Patient characteristics, including age, sex, smoking status, and responses to the Berlin questionnaire (BQ), were documented by trained interviewers using a computer-assisted standardized questionnaire. Patients with symptoms and characteristics consistent with OSA (hereinafter referred to as OSA) were identified using the BQ, and the general characteristics of the patients and their treatments as well as outcomes were recorded. The BQ is one of the most common questionnaires for OSA and has been validated in the primary care setting [11, 12]. Moreover, the sensitivity and specificity of the BQ have been validated in the Chinese population [13]. Body mass index (BMI) and blood pressure were measured with the patient in light indoor clothing and without shoes, by trained, licensed nurses of the PLA General Hospital. Blood samples were collected using a vacutainer tube. Fasting plasma glucose, triglyceride, total cholesterol, low-density lipoprotein cholesterol, high-density lipoprotein cholesterol, and markers of liver, renal, and cardiac function were routinely evaluated in a blinded manner in the PLA General Hospital Laboratory. The intra- and postoperative medications administered to the patients were recorded by the operating room nurses.

Conventional cardiovascular disease risk factors were defined as hypertension, obesity, diabetes, hyperlipidemia, and smoking [14]. Hypertension, diabetes, and hyperlipidemia were diagnosed by two associate chief physicians of the PLA General Hospital. Obesity was defined as a BMI ≥ 28 kg/m^2^, using the criteria for Asian people [15, 16]. A smoker was defined as a person who had smoked daily for at least 6 months during their lives [17]. Participants were divided into three groups according to the number of conventional cardiovascular disease risk factors present (≤1, 2, or ≥3 factors).

### Definitions and endpoints

Stent thrombosis was diagnosed based on the Academic Research Consortium specifications for probable or definite stent thrombosis [18]. Stent thrombosis was categorized as early (0–30 days after PCI), late (>30 days), and very late (>12 months). EST was further divided into acute (<24 h) and subacute (1–30 days). We only included patients with definite stent thrombosis detected on coronary angiography while in hospital.

### Statistical analysis

SPSS version 19.0 was used for data analysis. The significance level for all tests was set at a two-tailed α value of 0.05. The differences in means and proportions were tested using the paired *t*-test and chi-square test, respectively. Conditional logistic regression models were used to identify the odds ratio (OR) for OSA in patients with stent thrombosis.

### Ethical considerations

The Committee for Medical Ethics of the Chinese PLA General Hospital examined and approved our study protocol, and before completing the questionnaire, each participant signed an informed consent form.

## Results

### Patient characteristics

The general characteristics of the patients have been presented in Table 1. Our study involved 23 patients (18 men, 5 women) in the case group and 92 patients (72 men, 20 women) in the control group. The overall ratio of women to men was 5:18. The average ages of the participants in the case and control groups were 60.87 ± 10.90 years and 59.32 ± 9.76 years, respectively. Age, sex, smoking status, and incidence of hypertension, diabetes, hyperlipidemia, atrial fibrillation, myocardial infarction, and stroke did not differ between the two study groups (*P* > 0.05 for all). However, emergency operation (*P* = 0.007) and postoperative tirofiban usage (*P* = 0.000) were significantly more common in the case group than in the control group. Statin usage was less common (*P* = 0.004) and the implanted stents were significantly longer (*P* < 0.001) in the case group than in the control group. OSA was detected in 39 patients, 14 in the case group and 25 in the control group.

**Table 1:** General characteristics of the case and control groups

Characteristic	Case (*n* = 23)	Control (*n* = 92)	*P*-value
Age (years)	60.87 ± 10.90	59.32 ± 9.76	0.502
BMI (kg/m^2^)	26.29 ± 3.26	25.36 ± 3.07	0.198
Gender (male)	18 (78.26%)	72 (78.26%)	1.000
Smoking	16 (69.56%)	45 (48.91%)	0.076
Hypertension	13 (56.52%)	56 (60.87%)	0.703
Obesity	5 (21.7%)	14 (15.2%)	0.451
Overweight	19 (82.6%)	65 (70.7%)	0.248
Diabetes	8 (34.78%)	28 (30.43%)	0.688
Hyperlipidemia	4 (17.39%)	24 (26.09%)	0.385
Atrial fibrillation	0 (0.00%)	3 (3.26%)	0.380
Myocardial infarction	6 (26.09%)	19 (20.65%)	0.572
Stroke	4 (17.39%)	0 (0.00%)	0.999
OSA	14 (60.87%)	21 (23.09%)	0.002
Emergency operation	8 (34.78%)	10 (10.87%)	0.007
Bivalirudin	3 (13.04%)	0 (0%)	0.990
Postoperative tirofiban	14 (60.87%)	15 (16.30%)	0.000
Heparin	1 (4.35%)	15 (16.30%)	0.138
Statin	18 (78.26%)	91 (98.91%)	0.004
Length of stent (mm)	67.22 ± 31.64	38.60 ± 20.40	<0.001

Values are shown as mean ± standard deviation (SD) or number and percentage

*BMI* body mass index; *OSA* obstructive sleep apnea

### Effect of OSA on EST

The crude OR for OSA with EST was 4.17 (95% confidence interval [CI]: 1.60–10.84; Table 2). After adjusting for age, sex, obesity, smoking status, hypertension, diabetes, hyperlipidemia, emergency operation, tirofiban usage, statin usage, and stent length, the OR for OSA remained significant. Participants with OSA had a 7.34 times (95% CI: 1.52–35.58) higher risk of developing EST than did participants without OSA.

**Table 2:** OR for OSA in patients with early stent thrombosis

	Case	OR (95% CI)	*P*-value
OSA (crude)			0.003
No	9/76	1	
Yes	14/39	4.17 (1.60–10.84)	
OSA (adjusted for age and sex)			0.003
No	9/76	1	
Yes	14/39	4.26 (1.63–11.15)	
OSA (adjusted for age, sex, obesity, smoking, hypertension, diabetes, and hyperlipidemia)			0.003
No	9/76	1	
Yes	14/39	5.29 (1.76–15.93)	
OSA (adjusted for age, sex, obesity, smoking, hypertension, diabetes, hyperlipidemia, emergency operation, tirofiban use, statin use, and stent length)			0.013
No	9/76	1	
Yes	14/39	7.34 (1.52–35.58)	

*OR* odds ratio; *CI* confidence interval; *OSA* obstructive sleep apnea

**Fig. 1:**
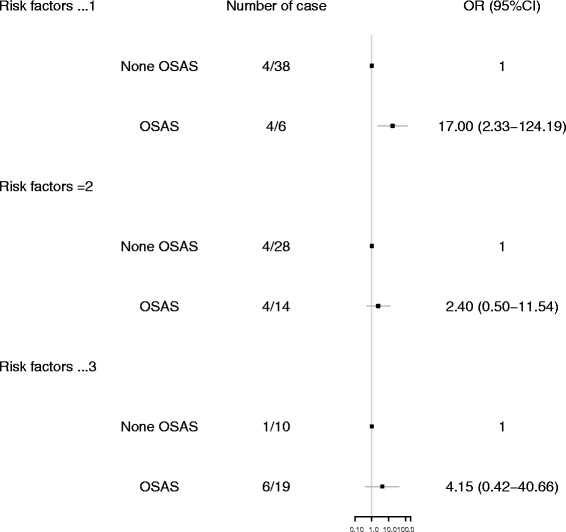
Odds ratio (OR) for obstructive sleep apnea (OSA) in patients with stent thrombosis based on the number of concomitant conventional cardiovascular risk factors

We also checked the association between OSA and EST in patients with different conventional cardiovascular risk factors (Fig. 1). In participants with no conventional risk factor or only one conventional risk factor, we found a significant association between OSA and EST (OR: 17.00, 95% CI: 2.33–124.19, *P* = 0.005). The effect of OSA on EST was not significant in participants with two risk factors or in participants with three or more risk factors (all ORs > 1.0).

## Discussion

The main findings of our study were that the prevalence of OSA was high in patients who developed EST after undergoing PCI and that OSA was an independent risk factor for EST. We found that 60.87% of patients with EST had OSA, which was significantly higher than the rate in the control group (23.09%) as well as that reported in the literature (48.3% or 57%) [19, 20]. Many studies have shown that patients with OSA are more likely to be male and have a high BMI [21]. A survey of adults aged 30–60 years from the general population found that the prevalence of OSA was 24% in men and 9% in women [3]. However, because of the relatively low number of female patients in the present study, the sex-specific difference in OSA prevalence was not analyzed.

The case and control groups had similar risks in terms of smoking, hypertension, diabetes, and hyperlipidemia, but operation type (emergency vs. non-emergency), tirofiban and statin usage, and stent length significantly differed between the two groups. These results are consistent with those of Jones et al. who found that hypertension, hypercholesterolemia, and diabetes were risk factors for stent thrombosis [22]. We therefore adjusted for obesity, smoking, hypertension, diabetes, hyperlipidemia, emergency operation, tirofiban use, statin use, and stent length in the conditional logistic regression models, and the analysis revealed that OSA was an independent risk factor for EST (Table 2). Further analysis of the relationship between OSA and EST based on the number of concomitant conventional risk factors showed a stronger relationship and a higher OR in patients with fewer conventional risk factors. This provides favorable evidence for OSA as a risk factor for EST.

The pathophysiology of stent thrombosis is multifactorial and depends on patient as well as treatment factors, such as vascular endothelial injury, excessive activation of platelets and the coagulation system, slow blood flow, systemic inflammatory response, type of operation, type of implant, and perioperative drug use [23]. Sleep apnea leads to recurrent, complete or partial obstruction of the upper airway, causing fluctuations in arterial blood oxygen saturation and arterial blood pressure [24]. Most of our patients were overweight, which is associated with excessive fat deposits around the upper airway, a smaller airway lumen, and increased collapsibility of the airway structures [25]. In obese patients, the root of the tongue can easily fall down and block the throat passage during sleep, leading to OSA. OSA can cause chronic intermittent hypoxia, and long-term hypoxia can damage multiple organs through oxidative stress and inflammation. Intermittent hypoxia and hypercapnia can also lead to considerable sympathetic nervous activation and vascular endothelial injury. This disrupts the balance between coagulation and fibrinolytic mechanisms, leading to further vascular endothelial injury and plaque formation. Thrombotic lesions and plaques in the coronary artery can become unstable and lead to emboli formation. Thus, OSA patients are in a hypercoagulable state [26], and have a high risk of stent thrombosis. In patients undergoing PCI, OSA has been associated with an increased 2-year composite MACE rate, mainly due to an increase in the periprocedural myocardial infarction rate [5]. Stent thrombosis is the main cause of postoperative acute myocardial infarction. Our study showed that OSA is an independent risk factor for stent thrombosis. This was further verified by the finding that in patients with few conventional cardiovascular risk factors, the contribution of OSA to stent thrombosis was more obvious. This discovery is worthy of clinical attention. The mechanism via which OSA increases the risk of stent thrombosis may be similar to that in chronic obstructive pulmonary disease (COPD)[27, 28]. Studies have shown that a screening test to identify those with negligible risk of undiagnosed COPD among patients with acute coronary syndrome is helpful to evaluate prognosis[29] and provide early treatment to reduce mortality risk[30]. These reports in combination with the results of our study suggest that early screening for OSA with the BQ may be necessary in patients with ischemic heart disease.

Stent size should be selected on the basis of lesion characteristics to ensure total attachment of the stent to the blood vessel wall and proper stent expansion. Techniques such as rotational atherectomy, cutting balloon angioplasty, intravenous ultrasonography, and optical coherence tomography can help ensure appropriate stent placement. Complete stent expansion and restoration of Thrombolysis in Myocardial Infarction grade-3 blood flow can reduce the risk of stent thrombosis. In addition to concerns about stent-implantation techniques and drug factors, identifying and closely monitoring patients who are at a high risk of stent thrombosis are of great importance.

### Study limitations

First, this was a case–control study, with some unavoidable biases. We used questionnaires that were answered by both patients and their families to minimize the impact of recall bias on the results of the study. Second, the incidence of stent thrombosis is low [1], and we only recruited patients with definite stent thrombosis; hence, the study sample was relatively small. Third, this is a single-center study based on clinical data from our hospital database. Fourth, OSA was diagnosed using only the BQ, as all our patients were scheduled to undergo surgery and most of them could not afford to undergo sleep-breathing monitoring. However, the BQ is the most common questionnaire for OSA and has been validated in the primary care setting [11, 12]. Furthermore, the sensitivity and specificity of the BQ have been validated in the Chinese population [13], and thus, the results are credible. Despite the above shortcomings, the relationship between OSA and postoperative stent thrombosis is still clear. We did not collect information on the treatments (such as continuous positive airway pressure) provided to the patients with symptoms and characteristics consistent with OSA, so we cannot report the effects of treatment on the prognosis of the patients in this study. An analysis of patient outcomes requires further research.

## Conclusion

OSA is an independent risk factor for EST in patients undergoing stent implantation. In addition, the effect of OSA on EST is more obvious in the presence of fewer conventional cardiovascular risk factors. Thus, it may be worthwhile to test for OSA in patients with few conventional cardiovascular risk factors.

## Abbreviations

BMI: Body mass index
BQ: Berlin questionnaire
COPD: Chronic obstructive pulmonary disease
EST: Early stent thrombosis
MACE: Major adverse cardiac events
OR: Odds ratio
OSA: Obstructive sleep apnea
PCI: Percutaneous coronary intervention
